# Effectiveness of Gas and Chimney Biomass Stoves for Reducing Household Air Pollution Pregnancy Exposure in Guatemala: Sociodemographic Effect Modifiers

**DOI:** 10.3390/ijerph17217723

**Published:** 2020-10-22

**Authors:** Laura M. Grajeda, Lisa M. Thompson, William Arriaga, Eduardo Canuz, Saad B. Omer, Michael Sage, Eduardo Azziz-Baumgartner, Joe P. Bryan, John P. McCracken

**Affiliations:** 1 Center for Health Studies, Universidad del Valle de Guatemala, Guatemala City 01015, Guatemala; ecanuz@gmail.com (E.C.); jmccracken@ces.uvg.edu.gt (J.P.M.); 2 Nell Hodgson Woodruff School of Nursing, Emory University, Atlanta, GA 30322, USA; lisa.thompson@emory.edu; 3 Regional Hospital, Ministry of Public Health Social Assistance of Guatemala, Quetzaltenango 09001, Guatemala; wear65@hotmail.com; 4 Yale Institute for Global Health, Schools of Public Health & Medicine, Yale University, New Haven, CT 06510, USA; saad.omer@yale.edu; 5 Division of Environmental Hazards and Health Effects, Centers for Disease Control and Prevention, Atlanta, GA 30341, USA; mikesage44@live.com; 6 Influenza Division, Centers for Disease Control and Prevention, Atlanta, GA 30341, USA; eha9@cdc.gov; 7 Division of Global Health Protection, Centers for Disease Control and Prevention, Atlanta, GA 30341, USA; joe.p.bryan@gmail.com; 8 Centers for Disease Control and Prevention, Central American Regional Office, Guatemala City 01015, Guatemala

**Keywords:** liquefied petroleum gas, biomass chimney stove, particulate matter, PM_2.5_, personal exposure, pregnancy, household air pollution

## Abstract

Household air pollution (HAP) due to solid fuel use during pregnancy is associated with adverse birth outcomes. The real-life effectiveness of clean cooking interventions has been disappointing overall yet variable, but the sociodemographic determinants are not well described. We measured personal 24-h PM_2.5_ (particulate matter <2.5 µm in aerodynamic diameter) thrice in pregnant women (*n* = 218) gravimetrically with Teflon filter, impactor, and personal pump setups. To estimate the effectiveness of owning chimney and liquefied petroleum gas (LPG) stoves (i.e., proportion of PM_2.5_ exposure that would be prevented) and to predict subject-specific typical exposures, we used linear mixed-effects models with log (PM_2.5_) as dependent variable and random intercept for subject. Median (IQR) personal PM_2.5_ in µg/m^3^ was 148 (90–249) for open fire, 78 (51–125) for chimney stove, and 55 (34–79) for LPG stoves. Adjusted effectiveness of LPG stoves was greater in women with ≥6 years of education (49% (95% CI: 34, 60)) versus <6 years (26% (95% CI: 5, 42)). In contrast, chimney stove adjusted effectiveness was greater in women with <6 years of education (50% (95% CI: 38, 60)), rural residence (46% (95% CI: 34, 55)) and lowest SES (socio-economic status) quartile (59% (95% CI: 45, 70)) than ≥6 years education (16% (95% CI: 22, 43)), urban (23% (95% CI: −164, 42)) and highest SES quartile (−44% (95% CI: −183, 27)), respectively. A minority of LPG stove owners (12%) and no chimney owner had typical exposure below World Health Organization Air Quality guidelines (35 μg/m^3^). Although having a cleaner stove alone typically does not lower exposure enough to protect health, understanding sociodemographic determinants of effectiveness may lead to better targeting, implementation, and adoption of interventions.

## 1. Introduction

The Global Burden of Disease Comparative Risk Assessment estimates that approximately 3.2% of the global total lost disability-adjusted life-years (DALYs) in 2016 were attributed to household air pollution (HAP). In Guatemala, HAP was responsible for an estimated 3.4% of DALYs [1]. HAP is generated through incomplete combustion of solid fuels used for cooking, resulting in the emission of pollutants, including PM_2.5_ (particulate matter <2.5 µm in aerodynamic diameter). Global estimates of 24-h personal exposure to PM_2.5_ for women using solid fuels in open fires had a pooled mean of 267 µg/m^3^ and a pooled standard deviation of 297 µg/m^3^ [2]. In contrast, the WHO air quality guidelines (WHO-AQG) recommend an interim annual mean target for PM_2.5_ ≤ 35 μg/m^3^.

Women of reproductive age are highly exposed to HAP because of their traditional role in cooking in many cultures [3]. Pregnancy is a vulnerable period because exposure to HAP is associated with adverse birth outcomes, such as low birth weight, stillbirth, and preterm birth [4,5,6,7,8,9]. Pregnancy might change cooking behaviors and time spent in the kitchen over the course of the pregnancy as other household members assume cooking roles [10]; therefore, exposure in non-pregnant women might generalize to pregnant women. However, only a few studies of personal exposure to PM_2.5_ during pregnancy have collected or are planned to collect repeated measures to estimate typical exposures [11,12,13,14].

Interventions to reduce HAP through cleaner stoves can lower PM_2.5_ concentrations. In a systematic review of before and after studies evaluating chimney stove effectiveness, the weighted mean percent reduction in personal exposure was 55% but range from 19% to 87% [15]. Liquefied petroleum gas (LPG) stoves have lower emissions and higher efficacy to reduce HAP under ideal adoption and use [12,16,17,18]. An intervention study aiming at estimating effectiveness conducted in Guatemala showed a 45% reduction in 24-h PM_3.5_ concentrations for LPG compared to the open fire [19]. Moreover, observational studies had reported percent differences in personal HAP exposure associated with LPG use ranging from 33% to 25% [20,21,22].

Although intervention with cleaner stoves reduces exposure to HAP, most do not typically reduce PM_2.5_ to levels low enough to meet WHO-AQG thought to protect health. Stove effectiveness and post-intervention exposure levels are heterogeneous, suggesting incomplete and variable adoption of cleaner technologies among population subgroups [15]. The level of stove adoption is influenced by social, geographic, financial, or individual factors [23,24,25,26]. Understanding the effect modification by sociodemographic factors will highlight population subgroups varying in levels of effectiveness. This data can inform intervention programs on pregnant who will benefit more from stoves and pregnant women who will need more support to adopt the technology.

Estimating LPG stove effectiveness in specific populations is valuable for designing epidemiological studies of HAP. Future studies could use LPG ownership as a surrogate for exposure if substantial exposure differences are detected. Measuring personal exposure is costly for researchers and is cumbersome for study participants, especially pregnant women [27]. Quantitative data on exclusive LPG stove use requires intensive monitoring of stoves, either with temperature sensors or frequent in-home assessments [28]. Because of this, we explored whether asking a simple question about LPG stove ownership, something that is easy to collect, is associated with substantial exposure reductions in a setting where the concurrent use of multiple stoves is common. If true, then LPG ownership may be used as a proxy for exposure in large, well-designed observational epidemiological studies or program evaluations.

Given the paucity of evidence and wide variability the real-life effectiveness of clean stoves interventions to reduce HAP exposure during pregnancy, we used repeated measures of personal PM_2.5_ in a pregnancy cohort study to address the following aims: (1) estimate the effectiveness of LPG stove ownership and chimney biomass stove ownership in reducing average PM_2.5_ exposure, (2) determine whether sociodemographic characteristics modify the effectiveness of cleaner stoves, and (3) compare subject-specific typical exposures in Guatemalan women to WHO recommended limits by type of stove owned.

## 2. Materials and Methods

### 2.1. Study Site and Population

We conducted the Embarazo Seguro Bebe Sano (Safe Pregnancy Healthy Baby) prospective pregnancy cohort study during 2013–2015 in San Juan Ostuncalco and Concepción Chiquirichapa, two municipalities of Quetzaltenango, Guatemala. These villages are in the western highlands ranging between 2000 and 2300 m above sea level. The population belongs primarily to the indigenous Mam ethnic group. The study was powered to assess the effect of acute respiratory infections on low birth weight. However, its cohort design allows studying environmental, nutritional, and infectious risk factors for disease during pregnancy and infancy [29]. This cohort of 224 non-smoking, low-risk for obstetrical complications pregnant women aged between 18 and 40 years were enrolled at <20 weeks gestation (estimated by ultrasound as part of the screening for recruitment eligibility) while seeking prenatal care at primary care clinics. We followed women and their infants until six months postpartum. The Ethics Committee Review Board for the Center for Health Studies, Universidad del Valle de Guatemala, approved this study under the protocol number 068-08-2012. The Ethics Committee of Emory University under the protocol number IRB00061308. The US Centers for Disease Control and Prevention considered investigators non-engaged. All participants provided written informed consent.

Sociodemographic data were obtained through interviews when women were <20 weeks of gestation. We collected information about years of maternal education, language spoken in the household as a proxy for ethnicity, age, and crowding (>3 persons per bedroom). Households in the town center of Concepción Chiquirichapa were classified as urban and the surrounding villages as rural residences. The accumulated wealth was assessed based on ownership of radio, television, refrigerator, motorcycle, car, computer, clothes washer, and house. We computed a wealth score using principal component analysis and then classified participants in wealth quartiles [30].

### 2.2. Stove Use and Other Sources of HAP Exposure

Participants were visited twice, at <20 and again at 26 weeks of gestation, to observe the types of stoves owned (LPG, chimney, or open fire). Solid fuel stoves without chimneys or with broken chimneys were classified as open fire stoves. During the visit we also conducted an interview about biomass use. The interview included cooking location (inside the main house as opposed to outside), frequency of stove use and median hours per day spent cooking during the last week. For analysis, stove ownership and biomass use variables at the most recent interview were carried forward to observations at 32 weeks gestation when these questions were not asked (*n* = 175).

Other sources of HAP include air pollution generated by household fuel combustion for lighting, heating, smoking, burning trash, or use of a traditional wood-fired sauna bath (“temascal” in Spanish) for bathing [31]. Trained study field workers partially accounted for these additional sources of HAP by interviewing whether electricity, exposure to secondhand smoke (SHS) from another household member, and wood-fired saunas were in the homes.

### 2.3. Personal Exposure Assessment

We measured twenty-four-hour average concentrations of personal HAP exposure at <20 weeks gestation in 220 women, at 26 weeks in 188 women, and 32 weeks in 176 women (584 measurements in total). Pregnant women wore for 24 h while conducting their regular activities, an impactor (SKC Personal Modular Impactor, SKC Inc., Eighty Four, Washington, PA, USA) with a Teflon ^TM^ filter (Pall US, Exton, PA, USA) and a pump (Casella Cel Tuff, Casella US, Buffalo, NY, USA) in a harness. Impactors were cleaned with alcohol and wiped with delicate task wipers after each sampling. The pumps were calibrated with a rotameter (High Accuracy Flowmeter, FM-1050 series, Matheson Tri-Gas, Inc., Montgomeryville, Pennsylvania, PA, USA) to keep the airflow rate at 1.5 L/min, measured at the beginning and the end of each sample. Teflon ^TM^ filters used to collect particles were weighed before and after sampling using an analytical microbalance (MT-5, Mettler-Toledo Inc., Columbus, OH, USA) with ±1 μg readability in an atmosphere-controlled room. Weights were taken in duplicate. If they differed by >5 µg, a third measurement was taken. The 24-h average concentration of PM_2.5_ was calculated by dividing the net filter weight by the volume of air sampled (sampling time multiplied by average flow rate). A measure was considered invalid if the net filter weight was negative or >3 μg, the sampling duration was <21 h, or if the average flow rate was <1.35 L/min or greater than 1.65 L/min. We excluded from the analysis 20 (3%) invalid measurements: seven had invalid net filter weights, twelve had a short duration, and three had average flow rates out of range.

We evaluated compliance in wearing the monitors by observing if women were wearing the monitors when trained field workers returned to remove equipment after 24-h of personal exposure measurement. In 90% of measurements, women were using the monitor; 77% of women not using the monitor reported taking it off one hour before the team’s arrival.

Twenty-five field blank filters were exposed to room air while assembling and disassembling the personal exposure equipment. The average blank filter weight change was 5.8 µg (range = 2–12), which was statistically different from 0 (*T*-test *p*-value < 0.001). The average blank net weight was subtracted from all sample filter net weights before estimating PM_2.5_ concentration. Twenty-two duplicate PM_2.5_ measurements were collected with co-located monitors. The duplicate measures of log PM_2.5_ showed correlation (Pearson correlation = 0.97, 95% CI: 0.94–0.99).

The between- and within-subject variance of log-transformed PM_2.5_ and the intraclass correlation coefficient (ICC) was calculated using an intercept mixed-effects model stratified by category of stove ownership. The ICC for chimney stove ownership excluded owners of LPG stove. Confidence intervals were estimated using the ICC R package [32]. We performed a sensitivity analysis to assess whether between-subject variance may be increased because of population time trends in exposure and the spread of individual follow-up periods (mean ~4 months) over a more extended study period (15 months). To conduct sensitivity analysis, we adjusted for study day using a cubic spline with three degrees of freedom.

### 2.4. Estimation of Pregnancy Average Concentration for PM_2.5_

We estimated subject-specific pregnancy exposure to PM_2.5_ using three separate methods: single 24-h measure, a subject-specific mean of 2–3 repeated 24-h measures, and subject mean using best linear unbiased predictor (BLUP). The BLUP was calculated with an intercept mixed-effects model using the participant level as the random effect [33,34]. Separate models were estimated for each category of stove ownership, allowing the mean and variance to differ by stove category. We visually tested the mixed-model assumptions by plotting the standardized residuals vs. fitted values to evaluate variance homogeneity and a quantile-quantile plot of the residuals to assess normality. We did not find major deviations from model assumptions. We compare the three approaches in terms of the estimated proportion of women with exposure below the WHO-AQG Interim Target-1 for annual mean PM_2.5_ (35 µg/m^3^) [31].

### 2.5. Directed Acyclic Graph (DAG)

**Figure 1:**
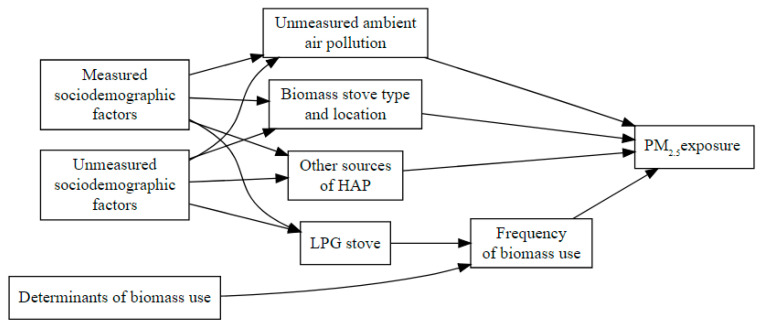
Directed acyclic graph of the hypothesized relationships between liquefied petroleum gas (LPG) stove ownership and exposure to personal fine particulate matter (PM_2.5_).

We use a DAG to determine the minimal sufficient adjustment set to estimate the average direct effect of LPG stove ownership on personal exposure to PM_2.5_ using DAGitty [35]. We hypothesized that sociodemographic factors (maternal age, maternal education, ethnicity, urban residence, wealth quartile, and crowding) had a direct effect on biomass stove type and location, LPG stove ownership, other sources of exposure to HAP (ownership of a wood-fired sauna bath, exposure to secondhand smoke, and having electricity), and ambient air pollution. We also assumed that exposure to PM_2.5_ was directly affected by biomass stove type and cooking location, frequency of biomass use, other sources of exposure to HAP, and ambient air pollution (Figure 1). The frequency of biomass use acts as an intermediate variable between LPG stove ownership and exposure to PM_2.5_ because the reduction in exposure to HAP is obtained by reducing the use of biomass, which is obtained by the greater use of LPG. Our DAG also considers residual confounding from unmeasured sociodemographic factors.

Sociodemographic factors included maternal education (≥6 years), maternal ethnicity (the spoken language in the household), maternal age, urban residence (Center of Concepción Chiquirichapa), wealth quartile, and crowding (>3 persons per bedroom). Biomass stove type and location included ownership of a chimney stove and whether the family cooks inside the main house as opposed to outside. Other sources of HAP included having electricity, exposure to secondhand smoke (SHS), and having a wood-fired sauna bath (temascal in Spanish). Determinants of biomass use include seasonality (dry season: months between November and April, rainy season: months between May and October), trimester of pregnancy, ownership of biomass chimney stove, maternal age, maternal education, ethnicity, urban residence, wealth quartile, and crowding.

The hypothesized determinants of stove effectiveness for reducing PM_2.5_ exposure were represented in the DAG as variables that might affect the frequency of biomass use. Investigated effect modifiers were seasonality (dry season: months between November and April, rainy season: months between May and October), trimester of pregnancy, ownership of biomass chimney stove, maternal age, maternal education, ethnicity, urban residence, wealth quartile, and crowding.

### 2.6. Analyses to Estimate the Effectiveness of Stove Ownership

The analysis consisted of crude and adjusted linear mixed-effects models using a log transformation of PM_2.5_ as the dependent variable, stove ownership as the independent variable, and a random intercept for subject. The model was adjusted for biomass stove type and biomass cooking location, other sources of exposure to HAP, and sociodemographic factors. The effectiveness of stove ownership was defined as the percent difference in personal PM_2.5_ calculated with the formula:

(1 − e^ß×LPG stove ownership^) × 100 (1)

The determinants of the effectiveness of stove ownership were investigated by adding an interaction term for each sociodemographic factor in the models. We visually tested the mixed-model assumptions by plotting the standardized residuals vs. fitted values to evaluate variance homogeneity and a quantile-quantile plot of the residuals to assess normality. We did not find major deviations from model assumptions. We used R: A Language and Environment for Statistical Computing, version 4.0.2 released on 2020-06-22 (R Foundation for Statistical Computing, Vienna, Austria) and RStudio: Integrated Development Environment for R, version 1.3.1073 released on 2020 (RStudio, PBC, Boston, MA, USA) for all analyses.

## 3. Results

We approached 637 women, of which 221 met all inclusion criteria and agreed to participate in the study; 218 had complete data on stoves and sociodemographic characteristics. At baseline, 27% (59/218) of pregnant women had an LPG stove. LPG stove owners had more years of education (*p* < 0.001), more frequently spoke Spanish (*p* < 0.001) rather than the Mam language, lived in an urban area (*p* < 0.001), were of higher wealth quartiles (*p* < 0.001) and had less household crowding (*p* < 0.01) than those without LPG stoves (Table 1). Although exposure to secondhand smoke from cigarettes (*p* = 0.237) or having electricity (*p* = 0.08) was similar between participants, owners of LPG stoves were less likely to have a wood-fired sauna (*p* < 0.01).

Our longitudinal data included 559 personal exposure measurements distributed among 211 women at <20 weeks, 178 at 26 weeks, and 170 at 32 weeks gestation. Twelve percent (27/218) of women had one, 19% (41/218) had two, and 69% (150/218) had three measurements. The gestational age during exposure measurement ranged from 6 to 39 weeks. Women with three repeated measures (*n* = 150, 69%) had a similar distribution of stoves owned, sociodemographic characteristics, and other sources of exposure to HAP compared with women with <3 repeated measures (*n* = 68, 31%). Women with three repeated measures were younger than women with <3 repeated measures (*p* = 0.015) (Table S1).

Study field workers observed that women had at least one biomass stove in almost all (97%) measurements, and 18% had two or three (Table 2). In most homes, the main stove was a chimney stove, although 28% of households used open fires. Open fire use was much more common (33%) among non-LPG owners than those with LPG stoves (15%). Most (99%) women used biomass fuel to prepare meals five or more times per week if they did not own LPG stoves vs. 66% of women with LPG stoves.

The geometric mean 24-h average PM_2.5_ exposure was 83 µg/m^3^ (95% CI: 78–89). Measurements recorded when field workers observed LPG stoves in the house had a geometric mean of 54 µg/m^3^ (95% CI: 49–60), and when LPG stoves were not observed, 98 µg/m^3^ (95% CI: 91–105). In comparison, when chimney stoves were observed, the geometric mean was 80 µg/m^3^ (95% CI: 74–87), and when chimney stoves were not observed, 146 µg/m^3^ (95% CI: 127–169). The overall ICC of exposure to PM_2.5_ was 0.51 (95% CI: 0.42–0.59). The ICC adjusted for study day was 0.52. We summarized the geometric mean, median, interquartile range, ICC, 95% confidence interval, and variance components of the log PM_2.5_ estimated for all measurements based on stove ownership in Table 3.

**Figure 2:**
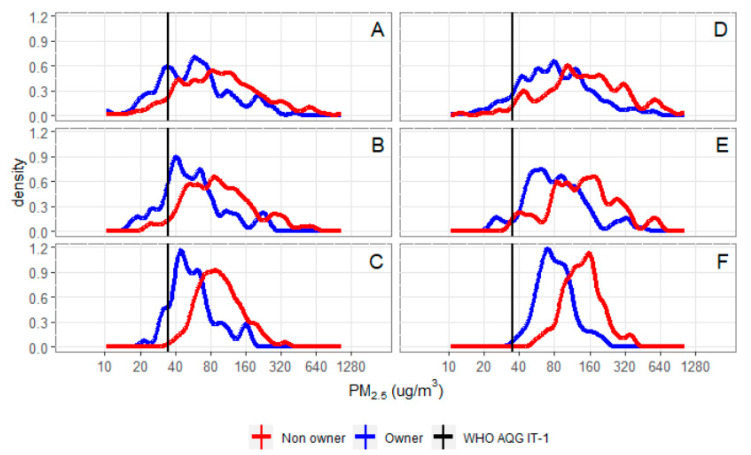
Distributions of alternative estimates of subject-specific exposure separated by LPG stove ownership (**A**–**C**) and chimney stove ownership, excluding LPG stove owners (**D**–**F**). Alternative estimates are single 24-h averages (**A**,**D**), subject means of 2–3 repeated 24-h measures (**B**,**E**), and subject mean using best linear unbiased predictor from the mixed model (**C**,**F**).

One quarter (39) of LPG stove owners met the WHO guideline if we used single measurements of 24-h average PM_2.5_ exposures (Figure 2A and Table 4). Using the subject mean of up to three 24-h average PM_2.5_ measures, 11 (19%) LPG stove owners met the WHO guideline (Figure 2B and Table 4). Only 12% (7/59) of LPG stove owners and none of the non-owners of an LPG stove met the WHO interim target of ≤35 µg/m^3^ (Figure 2C and Table 4). Using single measurements of 24-h average PM_2.5_ 27 (10%) of Chimney stove owners met the WHO-AQG (Figure 2D and Table 4). Using the subject mean of up to three 24-h average PM_2.5_ measures, 7 (6%) of chimney stove owners met the WHO guideline (Figure 2E and Table 4). None of chimney biomass stove owners had typical average personal exposures within WHO-AQG of ≤35 µg/m^3^ (Figure 2F and Table 4).

Personal exposure to HAP among pregnant women with an LPG stove was 38% lower (95% CI: 26–49%) than those without an LPG stove (Table 5). The effectiveness of the LPG stove was significantly greater in women with >6 years of education (49% (95% CI: 34–60%) than in women with ≤6 years was (26% (95% CI: 5–42%)). Among the subset without LPG stoves, chimney stove ownership was associated with a 43% (95% CI: 30–53%) effectiveness compared with open fires. In the effect modification analysis, the chimney stove effectiveness was significantly different within the residence (urban/rural) and maternal education years (Table 5).

## 4. Discussion

This study is one of the few observational longitudinal cohort studies to estimate the real-life effectiveness of both LPG and chimney stove ownership based on adjusted differences in personal PM_2.5_ in pregnant women. Moreover, we describe sociodemographic determinants of effectiveness in communities where both biomass and LPG fuel use are prevalent. Due primarily to the well-described use of multiple stoves, cooking devices, and different fuels in many settings [36,37], LPG stove effectiveness is uncertain and suspected to be heterogeneous. We found that LPG stove ownership was associated with an adjusted effectiveness of 38% compared with non-LPG stove owners. Additionally, women who owned an LPG stove had 33% lower exposures when owning chimney stoves and 52% when using open fires, suggesting that the effectiveness of LPG stoves might be modified by biomass stove ownership (*p*-value 0.083). Our estimates are similar to the 33% reduction in personal exposure to PM_2.5_ in pregnant women associated with using LPG for cooking instead of biomass in an effectiveness study conducted in rural Mexico [22]. A study conducted in Yunnan, China, contrasting personal exposure to PM_2.5_ in non-pregnant women primarily using improved fuels (LPG and electricity) with non-pregnant women primarily using biomass fuels reported a difference of personal exposure of 24% (91 µg/m^3^ vs. 119 µg/m^3^) [21]. Another study in the Yangtze River Delta in China reported a personal exposure to PM_2.5_ of 58 µg/m^3^ of PM_2.5_ in people using LPG and 77 µg/m^3^ in people using biomass, a 24% difference [20].

Among the subset without LPG stove, the effectiveness of a chimney biomass stove compared with non-chimney stove ownership was 43%. A meta-analysis of four before and after studies of personal PM_2.5_ estimated a weighted mean percent reduction of 55% ranging from 19% to 87% after chimney stove interventions [15]. These studies do not include pregnant women and measured efficacy rather than effectiveness.

We found that LPG stove adjusted effectiveness in women with ≥6 years of education was significantly higher than in women with <6 years. In contrast, when evaluating chimney stoves, women with <6 years of education, from rural areas and lower wealth quartiles experienced significantly higher adjusted effectiveness than ≥6 years of education, from urban areas and upper wealth quartiles, respectively. This finding suggests that more educated women will benefit more from LPG intervention programs. Education, independently of other sociodemographic factors, could drive the adoption of LPG through greater health literacy, in which women use health information to choose between different fuels [25]. However, in the absence of an LPG stove intervention, less educated, rural, and poorer women might benefit more from chimney stoves. Education, residence, wealth, and other sociodemographic variables have already been linked in population surveys of clean fuel as predictors of adoption [25,26,38,39,40]. Our study adds to the literature on how the distribution of determinants of adoption translates to exposure levels.

Temporal variability in exposure is known to cause measurement error when trying to estimate typical (months to years) exposure with short-term (24 or 48 h) measures. Although longitudinal models have previously been applied to assess the impact of this classical measurement error as a source of bias in exposure-response models, this method has not been used to improve estimates of the proportion of populations with typical average exposure below a target level. To estimate the typical prediction of exposure, we combined individual exposure data (personal exposure measurements) with group-level characteristics (LPG stove ownership) in a mixed-effects model and used the BLUP. This method takes advantage of the strengths of individual estimates and group-level estimates [41]. Predictions from mixed-effects models have smaller error variance in comparison to those from the short-term measures or the subject-specific average of repeated short-term measures, producing more precise estimates (Figure 2). As a result, our study demonstrates that fewer pregnant women breathed air with PM_2.5_ concentrations within the WHO recommended limits than if their exposures had been estimated directly from short term measures or subject averages. We found that pollutant reduction associated with LPG stove or chimney stove ownership were insufficient to achieve WHO-AQG. Only 12% (7/59) of pregnant women owning an LPG stove and no non-owners had a typical PM_2.5_ exposure ≤35 µg/m^3^ interim target. In the subset without the LPG stove, no women met this interim target.

The ICC of 51% found in this study suggests that three repeated short-term measurements of 24-h average exposure to PM_2.5_ provide a reliable estimate of subject-specific typical exposure. This ICC is higher than what was found in other studies [41,42,43,44,45]. High between-subject variation and low within-subject variation translates in enough exposure contrast between subjects and higher study power to conduct observational epidemiological studies. The ICC adjusted for study day was 0.52, suggesting that secular time trends and rolling recruitment over 16 months did not increase between-subject variance relative to within-subject variance.

Our study also demonstrates that LPG stove ownership indicates substantive lower personal exposure, even without accounting for actual stove use. The simplicity of collecting data on LPG stove ownership makes it a suitable exposure surrogate for population-based, large-scale-observational studies. Ownership measures the effect of LPG in everyday conditions; therefore, it is a measure of effectiveness representative of real circumstances and choices regarding fuel use. Observational studies of effectiveness, as opposed to controlled intervention studies of efficacy, are valuable to substantiate the impact of interventions in real-life conditions [46]. Then, to support identifying causal effects from an observational study, we adjusted effectiveness by possible confounders determined through a DAG.

This study has limitations. First, we do not know what proportion of women wore personal exposure equipment during the entire measurement period. However, only 10% were not using the monitor at the time the study team arrived at the house, and 77% of them reported taking it off in the last hour. If persons did not wear the equipment the entire day, their exposure measurements might be inaccurate (e.g., solely represent household PM_2.5_ rather than personal exposure). Second, we did not measure ambient air pollution and therefore cannot account for the proportion of exposure from ambient PM_2.5_ sources. However, based on DAG rules, by adjusting for the measured sociodemographic factors, we were able to partially control for confounding from ambient air pollution. Finally, we enrolled mainly ethnic Mam pregnant women seeking prenatal care at public primary care clinics in Guatemala. This population may not represent other ethnicities or women who do not receive prenatal care or receive it at private care clinics. In our study, 27% of women owned an LPG stove. In comparison, the 2018 Guatemalan national census reported LPG as the main cooking fuel in 23% of households in San Juan Ostuncalgo and 6% in Concepción Chiquirichapa [47].

## 5. Conclusions

LPG stove and chimney stove ownership in Guatemalan pregnant women was associated with a lower HAP pregnancy exposure. However, typical exposure levels only met WHO guidelines for a small minority of women owning LPG stove and none chimney owners, presumably because of concurrent biomass use. The effectiveness of LPG was higher in women with more education but the effectiveness of the chimney stove with less-educated, rural, and poor women. Understanding sociodemographic determinants of effectiveness may lead to better targeting, implementation, and adoption of interventions.

## Figures and Tables

**Table 1:** Baseline sociodemographic and household environmental characteristics among liquefied petroleum gas (LPG) stove owners and non-owners.

Sociodemographic or Environmental Characteristics	LPG Stove *n* = 59	No LPG Stove *n* = 159
Maternal Age Group, Years, *n* (%)		
18 to 20	11 (19)	41 (26)
21 to 30	35 (59)	80 (50)
31 to 40	13 (22)	38 (24)
Education, Years, Median (IQR ^1^)	9 (6–10)	4 (2–6)
Spanish Spoken in Household, *n* (%)	30 (51)	23 (15)
Urban Residence ^2^, *n* (%)	16 (27)	12 (8)
Wealth Quartile, *n* (%)		
Low	10 (17)	55 (35)
Low–Medium	7 (12)	55 (35)
Medium–High	13 (22)	26 (16)
High	29 (49)	23 (14)
Crowding, *n* (%) ^3^	11 (19)	66 (42)
Wood-Fired Sauna Bath, *n* (%)	50 (85)	153 (96)
Secondhand Smoke, *n* (%)	17 (29)	32 (20)
Electricity, *n* (%)	57 (97)	139 (87)

^1^ Interquartile range. ^2^ Residence was classified as urban or rural. ^3^ >3 persons per bedroom in a household.

**Table 2:** Characteristics of biomass stove use among overall personal exposure measures and by liquefied petroleum gas (LPG) stove ownership.

Characteristics of Biomass Use	Overall 559 (100%)	LPG Stove 149 (27%)	No LPG Stove 410 (73%)
Number of Biomass Stoves			
0	14 (3)	14 (9)	0 (0)
1	442 (79)	116 (78)	326 (80)
2 or 3	103 (18)	19 (13)	84 (20)
Biomass Stove Types ^1^			
Chimney Stove	390 (70)	113 (76)	277 (68)
Open Fire Stove	236 (42)	35 (23)	201 (49)
Main Biomass Stove			
Chimney Stove	387 (69)	113 (76)	274 (67)
Open Fire Stove	158 (28)	22 (15)	136 (33)
Frequency of Biomass Use			
≥5 Times per Week	502 (90)	98 (66)	404 (99)
<5 Times per Week	43 (8)	37 (25)	6 (1)
Cooks Inside Main House with Biomass	172 (31)	61 (41)	111 (27)
Median Hours/Day Cooking with Biomass (IQR ^2^)	3.0 (2.0–4.0)	3.0 (1.8–4.0)	3.0 (3.0–4.0)

^1^ Owned stoves do not add to 100% because households may have >1 stove. ^2^ Interquartile range.

**Table 3:** Description of the distribution of personal 24-h average PM_2.5_ (µg/m^3^) exposure overall, by liquefied petroleum gas (LPG) ownership, and by biomass chimney stove ownership.

Descriptors of Personal Exposure	Overall	LPG Stove	No LPG Stove	Chimney Stove ^3^	No Chimney Stove ^3^
Subjects	218	59	159	118	60
Measures	559	149	410	277	133
Minimum	10	10	11	11	13
Median (IQR ^1^)	79 (47, 137)	55 (34, 79)	96 (56, 160)	78 (51, 125)	148 (90, 249)
Geometric Mean (95% CI)	83 (78, 89)	54 (49, 60)	98 (91, 105)	80 (74, 87)	146 (127, 169)
Maximum	1052	284	1052	585	1052
Between-Participant Variance	0.33	0.20	0.29	0.18	0.26
Within-Participant Variance	0.32	0.23	0.33	0.29	0.43
ICC ^2^ (95% CI)	0.51 (0.42–0.59)	0.46 (0.27–0.62)	0.46 (0.36–0.56)	0.39 (0.25–0.52)	0.37 (0.16–0.56)

^1^ Interquartile range. ^2^ Intraclass correlation coefficients. ^3^ Population subset without LPG stove.

**Table 4:** Comparison of alternatives estimates of subject-specific pregnancy exposures by LPG stove ownership and chimney stove ownership.

Estimate of Exposure	Ownership of Stove	LPG Stove		Chimney Stove ^1^	
		Mean (SD) µg/m^3^	*n* Meeting AQG (%)	Mean (SD) µg/m^3^	*n* Meeting AQG (%)
24-h Averages ^2^	Owners	76 (64)	39 (25)	105 (89)	27 (10)
*n* = 559	Non-owner	133 (130)	34 (8)	192 (169)	5 (3)
Subject Mean ^3^	Owners	56 (51)	11 (19)	81 (75)	7 (6)
*n* = 218	Non-owner	96 (101)	7 (4)	138 (123)	1 (2)
Typical Exposures ^4^	Owners	57 (33)	7 (12)	81 (34)	0 (0)
*n* = 218	Non-owner	96 (53)	0 (0)	139 (61)	0 (0)

^1^ Population subset without LPG stove. ^2^ Single 24-h averages. ^3^ Subject-specific mean of 2–3 repeated 24-h measures. ^4^ Subject mean using the best linear unbiased predictor from mixed models. AQC World Health Organization Air Quality Guidelines interim target 1 (≤35 µg/m^3^).

**Table 5:** Determinants of the effectiveness of LPG and chimney stove ownership on the reduction of personal exposure to PM_2.5_ in pregnant women (*n* = 218).

Determinant	LPG Stove Ownership			Chimney Stove Ownership ^1^		
	*n*	% Effectiveness (95% CI)	Interaction *p-*Value ^2^	*n*	% Effectiveness (95% CI)	Interaction *p-*Value ^3^
All	559	38 (25, 49)				
Biomass Stove						
Chimney Stove	390	33 (18, 46)				
Open fire stove	169	52 (32, 65)	0.083	410	43 (31, 53)	
Season						
Rainy Season	316	37 (23, 49)		223	40 (24, 52)	
Dry Season	243	39 (22, 52)	0.849	187	48 (32, 59)	0.355
Residence						
Urban	69	34 (−4, 55)		37	23 (−164, 42)	
Rural	490	39 (25, 50)	0.732	373	46 (34, 55)	0.039
Spoken Language						
Spanish	135	31 (7, 50)		61	44 (−15, 73)	
Non-Spanish	424	41 (26, 53)	0.419	349	43 (30, 53)	0.945
Gestational Age						
1st Trimester	80	39 (14, 56)		58	43 (19, 61)	
2nd Trimester	169	27 (5, 44)	0.379	126	42 (23, 56)	0.921
3rd Trimester	299	40 (25, 52)	0.918	218	47 (33, 59)	0.704
Wealth Quartile						
Low	168	52 (30, 67)		136	59 (45, 70)	
Low–Medium	154	31 (−6, 55)	0.200	135	43 (22, 59)	0.142
Medium–High	104	29 (3, 51)	0.129	76	31 (−5, 54)	0.041
High	133	38 (18, 53)	0.260	63	−44 (−183, 27)	0.001
Persons per Bedroom						
>3	200	43 (18, 61)		177	46 (28, 59)	
≤3	359	37 (22, 48)	0.589	233	41 (23, 54)	0.672
Maternal Education, Years						
≤6	379	26 (5, 42)		304	50 (38, 60)	
>6	180	49 (34, 60)	0.029	106	16 (22, 43)	0.019
Maternal Age, Years						
18 to 20	144	34 (7, 53)		114	36 (10, 54)	
21 to 30	281	44 (29, 57)	0.379	194	50 (34, 63)	0.240
31 to 40	134	29 (0, 49)	0.776	102	39 (9, 59)	0.839

All models were adjusted for sociodemographic factors: maternal education (>6 years); maternal ethnicity (spoken language in the household); maternal age; urban residence (Center of Concepción Chiquirichapa); wealth quartile; crowding (>3 persons per bedroom), other sources of HAP (having electricity, exposure to secondhand smoke (SHS), having a sauna bath (*temascal* in Spanish)), and chimney biomass stove and cooking location (whether family cooks inside the main house as opposed to outside).^1^ Restricted to non-LPG owners. ^2^ This *p*-value corresponds to the interaction term between LPG stove ownership and the select sociodemographic factor. ^3^ This *p*-value corresponds to the interaction term between chimney stove ownership and the select sociodemographic factor.

## Supplementary Materials

The following are available online at https://www.mdpi.com/1660-4601/17/21/7723/s1, Table S1: Biomass use, baseline sociodemographic and household environmental characteristics by the number of repeated personal exposure measurements of PM_2.5_.

## References

[1] GBD (2017). Global, regional, and national comparative risk assessment of 84 behavioural, environmental and occupational, and metabolic risks or clusters of risks, 1990–2016: A systematic analysis for the Global Burden of Disease Study 2016. Lancet.

[2] Balakrishnan K., Mehta S. (2014). Population Levels of Household Air Pollution and Exposures.

[3] Balakrishnan K., Ghosh S., Ganguli B., Sambandam S., Bruce N., Barnes D.F., Smith K.R. (2013). State and national household concentrations of PM_2.5_ from solid cookfuel use: Results from measurements and modeling in India for estimation of the global burden of disease. Environ. Health.

[4] Boy E., Bruce N., Delgado H. (2002). Birth weight and exposure to kitchen wood smoke during pregnancy in rural Guatemala. Environ. Health Perspect..

[5] Tielsch J.M., Katz J., Thulasiraj R.D., Coles C.L., Sheeladevi S., Yanik E.L., Rahmathullah L. (2009). Exposure to indoor biomass fuel and tobacco smoke and risk of adverse reproductive outcomes, mortality, respiratory morbidity and growth among newborn infants in south India. Int. J. Epidemiol..

[6] Thompson L.M., Bruce N., Eskenazi B., Diaz A., Pope D., Smith K.R. (2011). Impact of reduced maternal exposures to wood smoke from an introduced chimney stove on newborn birth weight in rural Guatemala. Environ. Health Perspect..

[7] Amegah A.K., Quansah R., Jaakkola J.J. (2014). Household air pollution from solid fuel use and risk of adverse pregnancy outcomes: A systematic review and meta-analysis of the empirical evidence. PLoS ONE.

[8] Pope D.P., Mishra V., Thompson L., Siddiqui A.R., Rehfuess E.A., Weber M., Bruce N.G. (2010). Risk of low birth weight and stillbirth associated with indoor air pollution from solid fuel use in developing countries. Epidemiol. Rev..

[9] Balakrishnan K., Ghosh S., Thangavel G., Sambandam S., Mukhopadhyay K., Puttaswamy N., Sadasivam A., Ramaswamy P., Johnson P., Kuppuswamy R. (2018). Exposures to fine particulate matter (PM_2.5_) and birthweight in a rural-urban, mother-child cohort in Tamil Nadu, India. Environ. Res..

[10] Shezi B., Jafta N., Naidoo R.N. (2020). Exposure assessment of indoor particulate matter during pregnancy: A narrative review of the literature. Rev. Environ. Health.

[11] Wu J., Xiao X., Li Y., Yang F., Yang S., Sun L., Ma R., Wang M.C. (2020). Personal exposure to fine particulate matter (PM_2.5_) of pregnant women during three trimesters in rural Yunnan of China. Environ. Pollut..

[12] St Helen G., Aguilar-Villalobos M., Adetona O., Cassidy B., Bayer C.W., Hendry R., Hall D.B., Naeher L.P. (2015). Exposure of pregnant women to cookstove-related household air pollution in urban and periurban Trujillo, Peru. Arch. Environ. Occup. Health.

[13] Clasen T., Checkley W., Peel J.L., Balakrishnan K., McCracken J.P., Rosa G., Thompson L.M., Barr D.B., Clark M.L., Johnson M.A. (2020). Design and Rationale of the HAPIN Study: A Multicountry Randomized Controlled Trial to Assess the Effect of Liquefied Petroleum Gas Stove and Continuous Fuel Distribution. Environ. Health Perspect..

[14] Wylie B.J., Kishashu Y., Matechi E., Zhou Z., Coull B., Abioye A.I., Dionisio K.L., Mugusi F., Premji Z., Fawzi W. (2017). Maternal exposure to carbon monoxide and fine particulate matter during pregnancy in an urban Tanzanian cohort. Indoor Air.

[15] Pope D., Bruce N., Dherani M., Jagoe K., Rehfuess E. (2017). Real-life effectiveness of ‘improved’ stoves and clean fuels in reducing PM_2.5_ and CO: Systematic review and meta-analysis. Environ. Int..

[16] Naeher L.P., Leaderer B.P., Smith K.R. (2000). Particulate matter and carbon monoxide in highland Guatemala: Indoor and outdoor levels from traditional and improved wood stoves and gas stoves. Indoor Air.

[17] Johnson M.A., Chiang R.A. (2015). Quantitative Guidance for Stove Usage and Performance to Achieve Health and Environmental Targets. Environ. Health Perspect..

[18] Siddiqui A.R., Lee K., Bennett D., Yang X., Brown K.H., Bhutta Z.A., Gold E.B. (2009). Indoor carbon monoxide and PM_2.5_ concentrations by cooking fuels in Pakistan. Indoor Air.

[19] Albalak R., Bruce N., McCracken J.P., Smith K.R., De Gallardo T. (2001). Indoor respirable particulate matter concentrations from an open fire, improved cookstove, and LPG/open fire combination in a rural Guatemalan community. Environ. Sci. Technol..

[20] Hu R., Wang S., Aunan K., Zhao M., Chen L., Liu Z., Hansen M.H. (2019). Personal exposure to PM_2.5_ in Chinese rural households in the Yangtze River Delta. Indoor Air.

[21] Baumgartner J., Schauer J.J., Ezzati M., Lu L., Cheng C., Patz J., Bautista L.E. (2011). Patterns and predictors of personal exposure to indoor air pollution from biomass combustion among women and children in rural China. Indoor Air.

[22] Estevez-Garcia J.A., Schilmann A., Riojas-Rodriguez H., Berrueta V., Blanco S., Villasenor-Lozano C.G., Flores-Ramirez R., Cortez-Lugo M., Perez-Padilla R. (2020). Women exposure to household air pollution after an improved cookstove program in rural San Luis Potosi, Mexico. Sci. Total Environ..

[23] Hollada J., Williams K.N., Miele C.H., Danz D., Harvey S.A., Checkley W. (2017). Perceptions of Improved Biomass and Liquefied Petroleum Gas Stoves in Puno, Peru: Implications for Promoting Sustained and Exclusive Adoption of Clean Cooking Technologies. Int. J. Environ. Res. Public Health.

[24] Thompson L.M., Hengstermann M., Weinstein J.R., Diaz-Artiga A. (2018). Adoption of Liquefied Petroleum Gas Stoves in Guatemala: A Mixed-Methods Study. EcoHealth.

[25] Gould C.F., Urpelainen J. (2020). The Role of Education and Attitudes in Cooking Fuel Choice: Evidence from two states in India. Energy Sustain. Dev..

[26] Pope D., Bruce N., Higgerson J., Hyseni L., Ronzi S., Stanistreet D., Mbatchou B., Puzzolo E. (2018). Household Determinants of Liquified Petroleum Gas (LPG) as a Cooking Fuel in South West Cameroon. EcoHealth.

[27] Balakrishnan K., Sambandam S., Ghosh S., Mukhopadhyay K., Vaswani M., Arora N.K., Jack D., Pillariseti A., Bates M.N., Smith K.R. (2015). Household Air Pollution Exposures of Pregnant Women Receiving Advanced Combustion Cookstoves in India: Implications for Intervention. Ann. Glob. Health.

[28] Wilson D.L., Williams K.N., Pillarisetti A. (2020). An integrated sensor data logging, survey, and analytics platform for field research and its application in HAPIN, a multi-center household energy intervention trial. Sustainability.

[29] Weinstein J.R., Thompson L.M., Diaz Artiga A., Bryan J.P., Arriaga W.E., Omer S.B., McCracken J.P. (2018). Determining gestational age and preterm birth in rural Guatemala: A comparison of methods. PLoS ONE.

[30] Rutstein S.O. Steps to Constructing the New DHS Wealth Index. https://dhsprogram.com/programming/wealth%20index/Steps_to_constructing_the_new_DHS_Wealth_Index.pdf.

[31] WHO (2014). WHO Indoor Air Quality Guidelines: Household Fuel Combustion.

[32] Wolak M.E., Fairbairn D.J., Paulsen Y.R. (2015). Guidelines for Estimating Repeatability. Methods Ecol. Evol..

[33] Liu X.-Q., Rong J.-Y., Liu X.-Y. (2008). Best linear unbiased prediction for linear combinations in general mixed linear models. J. Multivar. Anal..

[34] Pinheiro J., Bates D. (2006). Mixed-Effects Models in S and S-PLUS.

[35] Textor J., vad der Zander B., Gilthorpe M.K., Liskiewicz M., Ellison G.T.H. (2016). Robust causal inference using directed acyclic graphs: The R package “dagitty”. Int. J. Epidemiol..

[36] Ruiz-Mercado I., Masera O. (2015). Patterns of stove use in the context of fuel-device stacking: Rationale and implications. EcoHealth.

[37] Thompson L.M., Diaz-Artiga A., Weinstein J.R., Handley M.A. (2018). Designing a behavioral intervention using the COM-B model and the theoretical domains framework to promote gas stove use in rural Guatemala: A formative research study. BMC Public Health.

[38] Puzzolo E., Pope D., Stanistreet D., Rehfuess E.A., Bruce N.G. (2016). Clean fuels for resource-poor settings: A systematic review of barriers and enablers to adoption and sustained use. Environ. Res..

[39] Lewis J.J., Pattanayak S.K. (2012). Who adopts improved fuels and cookstoves? A systematic review. Environ. Health Perspect..

[40] Heltberg R. (2005). Factors determining household fuel choice in Guatemala. Environ. Dev. Econ..

[41] McCracken J.P., Schwartz J., Bruce N., Mittleman M., Ryan L.M., Smith K.R. (2009). Combining individual- and group-level exposure information: Child carbon monoxide in the Guatemala woodstove randomized control trial. Epidemiology.

[42] Sanchez M., Mila C., Sreekanth V., Balakrishnan K., Sambandam S., Nieuwenhuijsen M., Kinra S., Marshall J.D., Tonne C. (2019). Personal exposure to particulate matter in peri-urban India: Predictors and association with ambient concentration at residence. J. Expo. Sci. Environ. Epidemiol..

[43] Dionisio K.L., Howie S.R., Dominici F., Fornace K.M., Spengler J.D., Donkor S., Chimah O., Oluwalana C., Ideh R.C., Ebruke B. (2012). The exposure of infants and children to carbon monoxide from biomass fuels in The Gambia: A measurement and modeling study. J. Expo. Sci. Environ. Epidemiol..

[44] Wafula E.M., Onyango F.E., Thairu H., Boleij J.S., Hoek F., Ruigewaard P., Kagwanja S., De Koning H., Pio A., Kimani E. (1990). Indoor air pollution in a Kenyan village. East Afr. Med. J..

[45] Saksena S., Prasad R., Pal R., Joshi V. (1992). Patterns of daily exposure to TSP and CO in the Garhwal Himalaya. Atmos. Environ. Part A Gen. Top..

[46] Peel J.L., Baumgartner J., Wellenius G.A., Clark M.L., Smith K.R. (2015). Are Randomized Trials Necessary to Advance Epidemiologic Research on Household Air Pollution?. Curr. Epidemiol. Rep..

[47] INE (2018). National Population and Household Census.

