# Supplementary material for: Effectiveness of Gas and Chimney Biomass Stoves for Reducing Household Air Pollution Pregnancy Exposure in Guatemala: Sociodemographic Effect Modifiers
Source: Int J Environ Res Public Health. 2020 Oct 22;17(21):7723. doi: 10.3390/ijerph17217723 (PMC7660060; doi:10.3390/ijerph17217723)
Supplement: Supplementary file 1 [file ijerph-17-07723-s001.pdf]

International Journal of
Environmental Research and Public Health

MDPI

Table S1. Biomass use, baseline sociodemographic and household environmental characteristics by the number of repeated personal exposure measurements of $PM_{2.5}$.

Variable	Women with 3 Repeated Measures *n* = 150 *n* (%)	Women with <3 Repeated Measures *n* = 68 *n* (%)	*p*-Value
Ownership of LPG Stove	43 (29)	16 (24)	0.531
Ownership of Chimney Stove	103 (69)	43 (63)	0.526
Open Fire	73 (49)	33 (49)	1.000
Cooks with Biomass Inside the House	43 (29)	26 (38)	0.211
Maternal Age, Years			
18 to 20	43 (29)	9 (13)	
21 to 30	70 (47)	45 (66)	0.015
31 to 40	37 (25)	14 (21)	
Maternal Education, Years, Mean (SD)	5 (3)	5 (3)	0.829
Spanish Spoken in Household	38 (25)	15 (22)	0.725
Urban Residence	17 (11)	11 (16)	0.440
Socioeconomic Asset Score			
1st Quartile	46 (31)	18 (26)	
2nd Quartile	40 (27)	23 (34)	0.703
3rd Quartile	27 (18)	10 (15)	
4th Quartile	37 (25)	17 (25)	
Crowding (>3 Persons per Bedroom)	55 (37)	22 (32)	
Traditional Sauna Bath (Temascal)	142 (95)	61 (90)	0.246
Second Hand Smoke	31 (21)	18 (26)	0.438
Electricity	135 (90)	61 (90)	1.000
